# Vitamin B12 and Folate Levels During Pregnancy and Risk of Gestational Diabetes Mellitus: A Systematic Review and Meta-Analysis

**DOI:** 10.3389/fnut.2021.670289

**Published:** 2021-06-14

**Authors:** Li Wang, Yanping Hou, Dexia Meng, Li Yang, Xiang Meng, Feng Liu

**Affiliations:** Department of Obstetrics, Zaozhuang Maternal and Child Health Hospital, Zaozhuang, China

**Keywords:** gestational diabetes, pregnancy, hyperglycemia, nutrition, insulin resistance

## Abstract

**Background:** The role of vitamin B12 and folate levels with risk of gestational diabetes mellitus (GDM) is unclear. The purpose of the current study was to conduct a systematic review and meta-analysis for assessing the relationship between vitamin B12 and folate concentrations during pregnancy and the risk of GDM.

**Methods:** PubMed, Embase, CENTRAL, and Ovid databases were searched up to 10th December, 2020 for all types of studies assessing the relationship. Qualitative and quantitative analysis of data was carried out.

**Results:** Twelve studies were included. Pooled serum vitamin B12 concentrations were found to be significantly lower in the GDM group as compared to the non-GDM group. No such difference was noted in serum folate levels. On pooled analysis of adjusted odds ratio's for risk of GDM with red blood cell (RBC) folate, serum folate, and vitamin B12 as continuous variables, no significant relationship was seen. On qualitative analysis, studies reported higher RBC folate levels with a significantly increased risk of GDM. Majority studies reported no relationship between serum folate and risk of GDM. Four of six studies reported a lowered risk of GDM with higher or normal vitamin B12 levels.

**Conclusion:** The association between vitamin B12 and folate levels during pregnancy and the risk of GDM is unclear. Limited number of studies indicate increased risk of GDM with higher RBC folate levels, but majority studies found no association between serum folate and risk of GDM. Based on available studies, the association between the risk of GDM with vitamin B12 deficiency is conflicting. There is a need for further large-scale studies from different regions worldwide to strengthen current evidence.

## Introduction

Gestational diabetes mellitus (GDM) is a common disorder during pregnancy affecting around 12.9% of pregnant females around the world (1). The disease is characterized by new-onset impaired glucose tolerance and insulin resistance during pregnancy which can lead to several adverse maternal and neonatal outcomes (2, 3). Women with GDM are at higher risk of preeclampsia, cesarean section, and post-partum diabetes mellitus while neonates have an increased risk of obesity and diabetes mellitus at a later age (3, 4). Therefore, recognition and modification of potential risk factors for GDM can significantly impact both maternal and neonatal health.

In recent years, the role of lifestyle and dietary modification in the prevention of GDM has been recognized (5). Amongst dietary factors, folate and vitamin B12 are essential nutrients required in early pregnancy, which are metabolically interlinked in one-carbon metabolism. The two are necessary for DNA methylation and production of nucleotides which in turn are needed for increased cellular replication and fetal growth (6). Specifically, folate is the donor of one-carbon units for the remethylation of homocysteine to methionine and then to S-adenosylmethionine (6). Folate along with Vitamin B12 as cofactor is necessary to maintain normal levels of homocysteine, as high levels of homocysteine are known to cause several pregnancy complications owing to its pro-inflammatory effect (7). Thus, both these nutrients (folate and Vitamin B12) are closely intertwined in this important metabolic function and deficiency of any of the two can potentially lead to pregnancy-related complications (7).

Folate supplements are recommended before and during early pregnancy for the prevention of neural tube defects (8). Supplemental folate intake to decrease nutritional deficiency has been made mandatory in more than 50 countries worldwide (9). With increased intake of folate, concerns about the negative implications of high folate levels have been raised. In the past decade, research has indicated a contrasting relationship between folate intake and GDM risk. Zhu et al. (10) in a prospective cohort study involving 1,938 females demonstrated a 2.25 times increased risk of GDM in women consuming daily folate supplements in the first trimester of pregnancy. On the other hand, in a recent study of 14,553 women, Li et al. (11) indicated a lowered risk of GDM in women with higher habitual intakes of supplemental folate before pregnancy.

Such contrasting data exists for the association between vitamin B12 and GDM as well. Studies have implicated lower vitamin B12 levels in pregnancy with adverse outcomes like obesity, insulin resistance, and GDM (12). On the other hand, a recent Chinese study on 1,058 pregnant females demonstrated an increased risk of GDM with higher vitamin B12 levels (13).

In this context, there is a lack of clarity on the association between vitamin B12 and folate levels during pregnancy and the risk of GDM. To the best of our knowledge, only one systematic review and meta-analysis has been published to date assessing the relationship between vitamin B12 and GDM risk (12). The review, however, did not assess the association of folate levels and could include only six studies. Therefore, the purpose of the current review was to systematically search the literature for studies assessing the relationship between vitamin B12 and folate concentrations during pregnancy and the risk of GDM and carry out a qualitative and quantitative analysis of data to present high-level evidence to patients as well as clinicians.

## Materials and Methods

### Inclusion Criteria

The review was conducted as per the PRISMA statement (Preferred Reporting Items for Systematic Reviews and Meta-analyses) (14). Inclusion criteria for the review were as follows:

(1) Prospective or retrospective cohort studies and cross-sectional studies reporting the incidence or prevalence of GDM based on concentrations of vitamin B12 and/or folate in pregnant women (2) Studies were to compare concentrations of vitamin B12 and/or folate in GDM patients with a control group of non-GDM women. (3) Studies assessing both serum folate, and red blood cell (RBC) folate levels were eligible for inclusion.

The following studies were excluded: (1) Studies reporting risk of GDM based on the intake of folate or vitamin B12 supplements and not on biochemical values. (2) Studies assessing the risk of GDM based on any other vitamins. (3) Studies without a control group. (4) Review articles, non-English language studies, case series and case reports. For studies reporting duplicate or overlapping data, the study with the larger sample size was to be included.

### Search Strategy

An electronic search was conducted in the PubMed, Embase, CENTRAL, and Ovid databases to identify relevant publications. All databases were screened from inception to 10th December, 2020. The search was conducted by two reviewers independent of each other. Keywords used in different combinations were: “vitamin B12,” “folic acid,” “folate,” “gestational diabetes,” “gestational,” and “hyperglycemia.” Supplementary Table 1 demonstrates the search strategy. Every search result was evaluated by the two reviewers independently, initially by their titles and abstracts and then by full texts of relevant publications. All full-texts were reviewed based on the inclusion and exclusion criteria and the article satisfying all the criteria was finally selected for this review. Any disagreements were resolved by discussion. To avoid any missed studies, the bibliography of included studies were hand searched for any additional references.

### Data Extraction and Risk of Bias Assessment

We prepared a data extraction form at the beginning of the review to extract relevant details from the studies. Details of the first author, publication year, study type, study location, sample size, age, and body mass index (BMI) of patients, testing protocol and criteria for GDM, serum folate, and vitamin B12 concentrations, and study outcomes were extracted. The outcome of interest was to assess the risk of developing GDM based on vitamin B12 and folate levels. We also compared the serum concentrations of vitamin B12 and folate between the GDM and non-GDM groups.

Since all included studies were cross-sectional or cohort studies, we assessed the quality of included studies using the risk of a bias assessment tool for non-randomized studies (RoBANS) (15). Two reviewers assessed each study for: selection of participants, confounding variables, intervention measurements, blinding of outcome assessment, incomplete outcome data, and selective outcome reporting.

### Statistical Analysis

“Review Manager” [RevMan, version 5.3; Nordic Cochrane Center (Cochrane Collaboration), Copenhagen, Denmark; 2014] was used for the meta-analysis. We initially pooled mean and standard deviation (SD) of serum concentrations of vitamin B12 and folate in the GDM and non-GDM groups to calculate the mean difference (MD) with 95% confidence intervals (CI). For studies not reporting mean and SD values, the same was calculated from the median and interquartile range (16). We also extracted multivariable-adjusted odds ratios (OR) for the risk of developing GDM based on serum concentrations of vitamin b12, serum folate, and RBC folate levels and pooled data using the generic inverse function of the meta-analysis software. For data that could not be pooled due to heterogeneous reporting, a descriptive analysis was carried out. A meta-analysis was conducted if at least three studies reported the same outcome on a homogenous scale. A random-effects model was preferred for the meta-analysis. The *I*^2^ statistic was used to assess inter-study heterogeneity. *I*^2^ values of 25–50% represented low, values of 50–75% medium, and more than 75% represented substantial heterogeneity. Funnel plots were used to assess publication bias.

## Results

**Figure 1:**
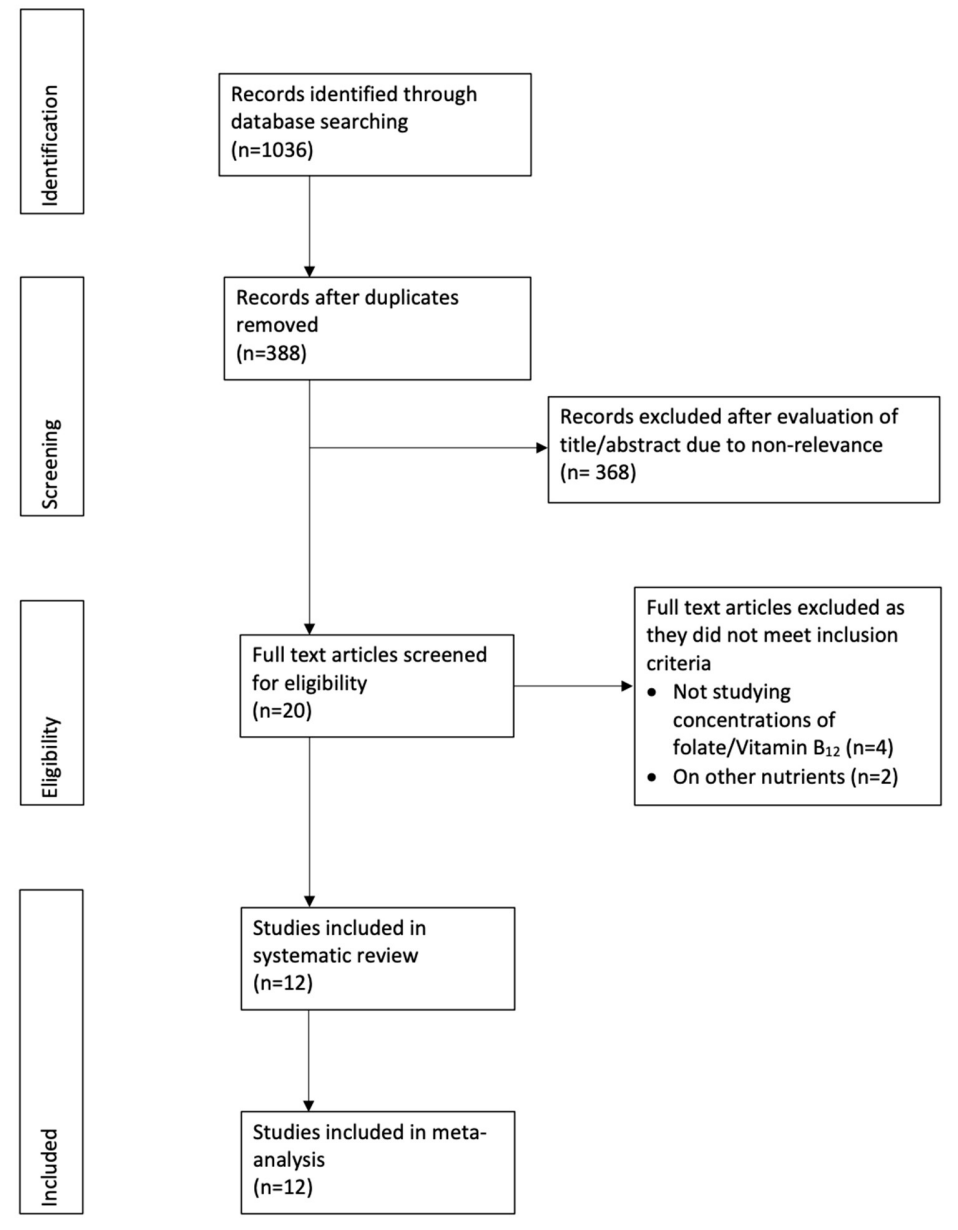
Study flow chart.

Figure 1 presents the study flow-chart. A total of 12 studies fulfilled the inclusion criteria and were included in the review (13, 17–27). Details of the included studies are presented in Table 1. The majority of the studies were prospective in nature. Four of the 12 studies were carried out in China, two in Turkey, while one each in the United Kingdom, India, Singapore, Italy, Poland, Australia, and New Zealand. The oral glucose tolerance test (OGTT) was used in all studies for diagnosing GDM, however, the diagnostic criteria varied. The sample size in the GDM group varied from 15 to 392 patients. The risk of bias analysis of included studies is presented in Supplementary Table 2.

**Table 1:** Characteristics of included studies.

**References**	**Location**	**Type**	**Test for GDM**	**Criteria for GDM**	**Testing period**	**Testing period for folate, vitamin B12**	**Sample size**	**Mean age (years)**	**Mean BMI (kg/m^**2**^)**	**Outcomes measured**
Jankovic-Karasoulos et al. (26)	Australia and New Zealand	Prospective cohort	Fasting glucose or OGTT	FPG ≥5.1 mmol/L or following an OGTT, a 2-h level of ≥8.5 mmol/L	Around 26 weeks	15 ± 1 week of gestation	GDM: 33	NR	NR	Serum folate, vitamin B12
							Control: 111			
Liu et al. (27)	China	Prospective cohort	75 g OGTT	FPG ≥ 5.1 mmol/L, 1-h plasma glucose ≥ 10.0 mmol/L, and 2-h plasma glucose ≥ 8.5 mmol/L	24–28 weeks	1st trimester	GDM: 67	30.5 ± 4	24.3 ± 3.6	RBC folate
							Control: 299	28.9 ± 3.5	22.4 ± 3.6	
Chen et al. (13)	China	Prospective cohort	75 g OGTT	FPG ≥ 5.1 mmol/L, 1-h plasma glucose ≥ 10.0 mmol/L, and 2-h plasma glucose ≥ 8.5 mmol/L	24–28 weeks	9–13 weeks of gestation	Total: 1,058 (180 with GDM)	30.2 ± 4	21 ± 2.8	RBC folate, serum folate, vitamin B12
Xie et al. (24)	China	Prospective cohort	75 g OGTT	FPG ≥ 5.5 mmol/L, 2-h plasma glucose ≥ 8 mmol/L	24–28 weeks	2nd trimester	GDM: 392	29 ± 3.2	24.3 ± 3.1	RBC folate
							Control: 1,890	27.9 ± 3.2	23.2 ± 2.7	
Li et al. (25)	China	Prospective cohort	75 g OGTT	FPG ≥ 5.1 mmol/L, 1-h plasma glucose ≥ 10.0 mmol/L, and 2-h plasma glucose ≥ 8.5 mmol/L	24–28 weeks	24–28 weeks	Total: 406 (90 with GDM)	29.4 ± 4.5	NR	Serum folate, vitamin B12
Lai et al. (23)	Singapore	Prospective cohort	75 g OGTT	FPG ≥ 7 mmol/L, 2-h plasma glucose ≥ 7.8 mmol/L	26–28 weeks	26–28 weeks	Total: 913 (164 with GDM)	NR	NR	Serum folate, vitamin B12
Sukumar et al. (22)	UK	Retrospective cohort	75 g OGTT	FPG ≥ 6.1 mmol/L, 2-h plasma glucose ≥ 7.8 mmol/L	26–28 weeks	2nd trimester	GDM: 143	31.4 ± 5.8	31.7 ± 7	Serum folate, vitamin B12
							Control: 201	29.6 ± 5.9	26.7 ± 7.1	
Krishnaveni et al. (20)	India	Retrospective cohort	100 g OGTT	FPG ≥ 7 mmol/L, 2-h plasma glucose ≥ 11.1 mmol/L	30 weeks	30 weeks	Total: 519 (35 with GDM)	24 (21–26)^*^	23.5 (20.5–26.9)^*^	Serum folate, vitamin B12
Idzior- Waluś et al. (21)	Poland	Prospective cohort	75 g OGTT	FPG ≥ 6.1 mmol/L, 2-h plasma glucose ≥ 7.8 mmol/L	26–32 weeks	26–32 weeks	GDM: 44	30.5 ± 6.6	27.8 ± 5.2	Serum folate, vitamin B12
							Control: 17	26.3 ± 4	25.6 ± 3.4	
Guven et al. (19)	Turkey	Cross-sectional	100 g OGTT	Two or more of the following plasma values must be met or exceeded: FPG 95 mg/dl, 1 h 180 mg/dl, 2 h 155 mg/dl and 3 h 140 mg/dl	24–28 weeks	24–28 weeks	GDM: 30	30 ± 4.3	29.2 ± 4.1	Serum folate, vitamin B12
							Control: 147	28.6 ± 3.4	27.9 ± 2.8	
Tarim et al. (18)	Turkey	Prospective cohort	50 g OGTT	Two or more of the following plasma values must be met or exceeded: FPG 95 mg/dl, 1 h 180 mg/dl, 2 h 155 mg/dl and 3 h 140 mg/dl	24–28 weeks	24–28 weeks	GDM: 28	32 ± 4	27.1 ± 2.1	Serum folate, vitamin B12
							Control: 210	26.8 ± 4.4	25.2 ± 2	
Seghieri et al. (17)	Italy	Cross-sectional	100 g OGTT	NR	24–28 weeks	24–28 weeks	GDM: 15	34.6 ± 3.1	26.7 ± 3.2	Serum folate, vitamin B12
							Control: 78	32.3 ± 3.7	26.3 ± 3.7	

*GDM, gestation diabetes mellitus; OGTT, oral-glucose tolerance test; NA, Not available; FPG, fasting plasma glucose; h, hour*.

* *Median (interquartile)*.

### Meta-Analysis

**Figure 2:**
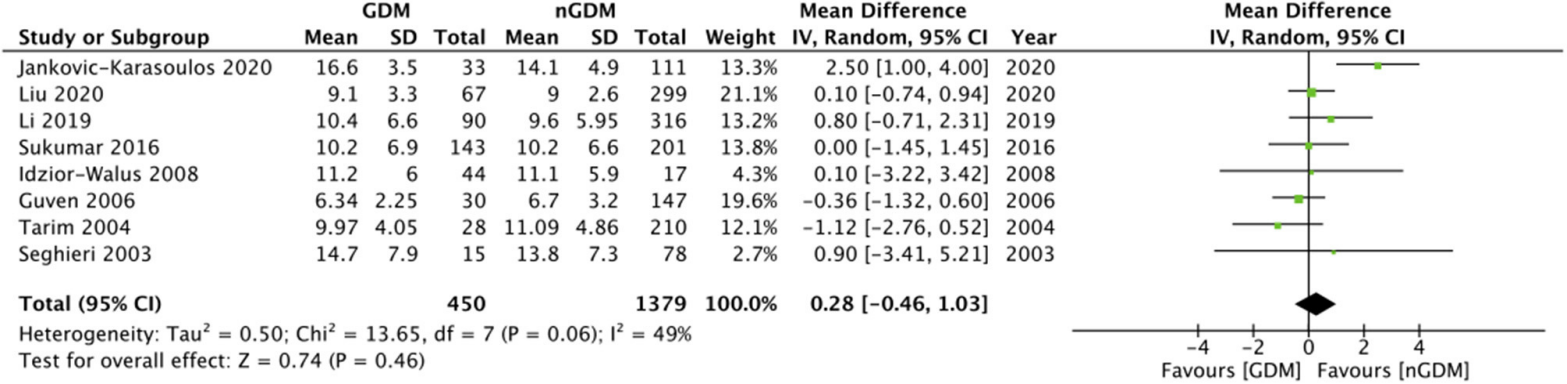
Meta-analysis of serum folate concentration during pregnancy between GDM and non-GDM groups.

**Figure 3:**
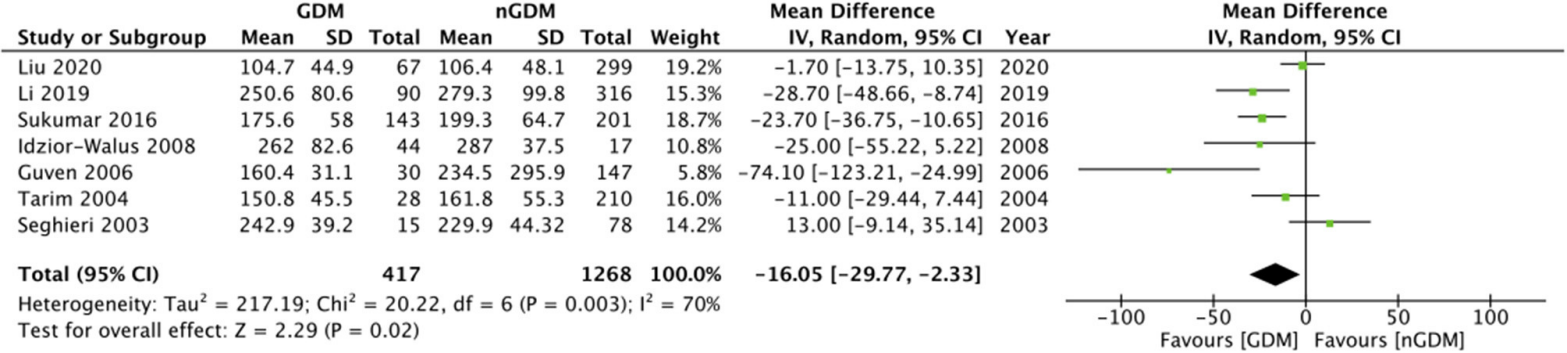
Meta-analysis of serum vitamin B12 concentration during pregnancy between GDM and non-GDM groups.

Comparing the serum folate concentration (ng/ml) between the GDM and non-GDM group, our pooled analysis indicated no difference between the study groups (MD: 0.28 95%CI: −0.46, 1.03 *I*^2^ = 49% *p* = 0.46) (Figure 2). However, serum vitamin B12 concentrations (pg/ml) were found to be significantly lower in the GDM group as compared to the non-GDM group (MD: −16.05 95%CI: −29.77, −2.33 *I*^2^ = 70% *p* = 0.02) (Figure 3). There was no evidence of publication bias in both the analyses (Supplementary Figures 1, 2).

**Figure 4:**
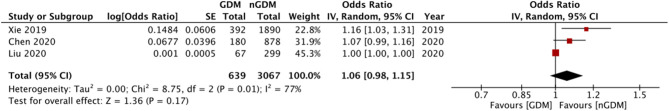
Meta-analysis of adjusted odds ratios assessing relationship between GDM and RBC folate (as continuous variable).

**Figure 5:**
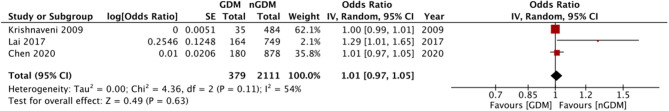
Meta-analysis of adjusted odds ratios assessing relationship between GDM and serum folate (as continuous variable).

**Figure 6:**
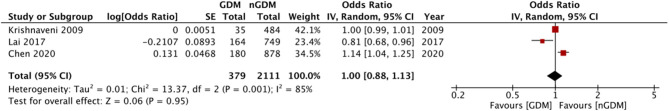
Meta-analysis of adjusted odds ratios assessing relationship between GDM and vitamin B12 (as continuous variable).

Multivariable adjusted ORs for risk of GDM were reported by eight of the 12 included studies. Details are presented in Table 2. Six of these studies reported an association between the risk of GDM with serum folate and vitamin B12 concentrations. Three studies reported data on the risk of GDM based on RBC folate levels. Only three studies reported adjusted ORs for risk of GDM with RBC folate, serum folate, and vitamin B12 as continuous variables. Pooled analysis demonstrated no statistically significant relation between RBC folate levels and risk of GDM (OR: 1.06 95%CI: 0.98, 1.15 *I*^2^ = 77% *p* = 0.17) (Figure 4). Similarly, no significant relationship was noted between serum folate levels (OR: 1.01 95%CI: 0.97, 1.05 *I*^2^ = 54% *p* = 0.63) (Figure 5) and vitamin B12 level (OR: 1.00 95%CI: 0.88, 1.13 *I*^2^ = 85% *p* = 0.95) (Figure 6) with risk of GDM. There was no evidence of publication bias in either analyses (Supplementary Figures 3–5).

**Table 2:** Multivariable adjusted Odds ratios from the included studies.

**References**	**Adjusted factors for multivariable analysis**	**Outcome**	**Adjusted OR (95% CI)**
Jankovic-Karasoulos et al. (26)	Age, BMI, smoking status	Vitamin B12 Every 50-unit increase	0.99 (0.83–1.18)
		Serum folate Every 5-unit increase	1.22 (0.93–1.59)
Liu et al. (27)	Age, physical activity, BMI, parity, family history of diabetes, use of folic acid supplements, HOMA-IR, C-reactive protein, hemoglobin, vitamin B12, and serum homocysteine	RBC folate Q1 <224.6 ng/ml Q2 224.6–285.9 ng/ml Q3 285.9–380,6 ng/ml Q4 ≥380.6 ng/ml As continuous variable	Reference 1.354 (0.530–3.456) 1.374 (0.546–3.455) 2.473 (1.013–6.037) 1.001 (1.000–1.002)
Chen et al. (13)	Age, preconceptional BMI, family history of diabetes, smoking exposure, and drinking status	RBC folate Q1 <400 ng/ml Q2 400–600 ng/ml Q3 ≥600 ng/ml As continuous variable	Reference 1.39 (0.94–2.04) 1.58 (1.03–2.41) 1.07 (0.99–1.15)
		Serum folate Q1 <13.9 ng/ml Q2 13.9–16 ng/ml Q3 ≥16 ng/ml As continuous variable	Reference 0.91 (0.58–1.44) 1.36 (0.94–1.99) 1.01 (0.97–1.05)
		Vitamin B12 Q1 <330 pg/ml Q2 330–429 pg/ml Q3 ≥429 pg/ml As continuous variable	Reference 1.49 (0.95–2.33) 2 (1.35–2.96) 1.14 (1.04–1.24)
Xie et al. (24)	Age, parity, BMI at enrolment	RBC folate Q1 <399.8 ng/ml Q2 399.8–570.2 ng/ml Q3 ≥570.2 ng/ml As continuous variable	Reference 2.17 (1.20–3.95) 2.76 (1.56–4.89) 1.16 (1.03–1.30)
Li et al. (25)	Age, ethnicity, education, parity, pre-pregnancy BMI, and family history of diabetes, folate (or vitamin B12)	Serum folate Q1 <6.9 ng/ml Q2 6.9–12.2 ng/ml Q3 ≥12.2 ng/ml	Reference 1.12 (0.59–2.13) 1.98 (1.0–3.90)
		Vitamin B12 Q1 <234 pg/ml Q2 234–306 pg/ml Q3 ≥306 pg/ml	Reference 0.51 (0.27–0.95) 0.30 (0.15–0.60)
Lai et al. (23)	Age, ethnicity, education, income, smoking, alcohol intake, physical activity, pre-pregnancy BMI, parity, family history of diabetes, and previous occurrence of GDM, folate (or vitamin B_6_ and B_12_)	Serum folate As continuous variable	1.29 (1.01, 1.60)
		Vitamin B12 As continuous variable	0.81 (0.68, 0.97)
Sukumar et al. (22)	Age, parity, ethnic origin, smoking, gestation of bloods, and serum folate (or vitamin B12)	Serum folate 3.1–18.7 ng/ml <3.1 ng/ml	Reference 0.89 (0.07, 11.38)
		Vitamin B12 203.3–489 pg/ml <203.3 pg/ml	Reference 2.05 (1.03, 4.10)
Krishnaveni et al. (20)	Age, socioeconomic status, religion, parity and family history of diabetes	Serum folate As continuous variable	1.0 (0.99, 1.0)
		Vitamin B_12_ 203.3–489 pg/ml <203.3 pg/ml As continuous variable	Reference 2.0 (1.1, 3.6) 1.0 (0.99, 1.0)

*OR, Odds ratio; CI, confidence interval; Q, quartile; HOMA-IR, Homeostasis model assessment- insulin resistance; BMI, Body mass index*.

### Descriptive Analysis-Folate Levels

There was a wide variation in the classification of quartile ranges for defining the association between the study variables and GDM risk. Hence, a meta-analysis could not be conducted, and instead, a descriptive analysis was carried out. For RBC folate levels, all three studies reported a significantly increased risk of GDM in the highest quartiles of their respective studies. Liu et al. (27) reported 2.473 times (95% CI: 1.013, 6.037) increased risk of GDM with RBC folate levels of ≥ 380.6 ng/ml. Similarly, Chen et al. (13) reported 1.58 times (95% CI: 1.03, 2.41) and Xie et al. (24) reported 2.76 times (95% CI: 1.56, 4.89) increased risk of GDM with RBC folate levels of ≥600 ng/ml and 570.2 ng/ml, respectively. Of the six studies assessing the relation between serum folate and GDM, four (13, 20, 22, 26) reported no statistically significant association between serum folate levels and risk of GDM. Li et al. (25) reported 1.98 times (95% CI: 1, 3.90) increased risk of GDM with serum folate levels ≥12.2 ng/ml, and Lai et al. (23) reported 1.29 times (95% CI: 1.01, 1.60) increased risk of GDM with an incremental increase in serum folate. Of the four studies not reporting adjusted ORs, none reported any statistically significant difference in serum folate levels between GDM and non-GDM groups. Similarly, no difference was noted in vitamin B12 levels, except for Seghieri et al. (17) which reported lower vitamin B12 levels in women with GDM.

### Descriptive Analysis-Vitamin B12 Levels

Of the six studies assessing the relationship between serum vitamin B12 levels and risk of GDM, only one study reported no statistically significant relationship between the two (26). Two studies reported a statistically significant increased risk of GDM with vitamin B12 deficiency. Krishnaveni et al. (20) and Sukumar et al. (22) reported two times increased risk of GDM with vitamin B12 levels <203.3 pg/ml. In line with these studies, Lai et al. (23) reported a significantly lowered risk of GDM with a per unit increase in vitamin B12 levels. Li et al. (25) also found a significantly reduced risk of GDM with vitamin B12 >234 pg/ml. Only Chen et al. (13) reported 2 times (95% CI: 1.35, 2.96) increased risk of GDM with vitamin B12 > 429 pg/ml.

## Discussion

Folate is an extremely important micronutrient for pregnant females due to its role in preventing birth defects (28). Given the evidence, the USA Public Health Service recommends daily supplementation of 0.4–0.8 mg of folic acid for all pregnant females (29). Studies have indicated that food fortification with folates significantly reduces the incidence of neural tube defects (28). However, with the widespread consumption of folate, especially in pregnant females, the potential adverse risks of high folate levels on both the mother and the child have caused concerns. Yajnik et al. (30) have demonstrated that high RBC folate and low vitamin B12 levels in mothers during pregnancy result in an increased risk of insulin resistance and higher adiposity in the offspring. Similar concerns have been raised for the risk of maternal GDM with preconceptional and periconceptional folate supplements but with conflicting results (10, 11). The contradictory results in literature can be partially attributed to the difference in timing of folate supplements, the dosage as well as on patient adherence. Furthermore, folate metabolism can vary amongst individuals leading to varying serum folate levels (31). Thus, it is better to assess the risk of GDM based on actual body folate concentrations rather than the intake of folate supplements. Thus, we excluded studies reporting associations between nutritional supplements and risk of GDM without measuring maternal folate or vitamin B12 levels.

Studies included in our review measured both sera as well as RBC folate levels. While serum folate levels are easily altered by folate intake or supplements, RBC folate levels correspond to long-term intake of folate supplementation or fortified foods (13, 32). WHO guidelines recommend that RBC folate concentrations be maintained above 400 ng/ml to achieve the greatest reduction of neural tube defects. However, as serum folate concentrations are rapidly altered by folate intake, no such threshold was provided for serum folate levels (33). In our analysis, we found no statistically significant difference in serum folate levels between GDM and non-GDM women. Four of the six studies reported no association between serum folate and risk of GDM. Also, on the meta-analysis of RBC folate and serum folate as continuous variables, we could not find any significant relationship between folate levels and the risk of GDM. However, all three studies measuring RBC folate levels found a significantly increased risk of GDM with higher RBC folate quartiles (ranging from >380.6 to 600 ng/ml). Thus, the results indicate that higher RBC folate levels are associated with increased risk of GDM, however, the association is non-linear. Given the limited number of studies which were all conducted in a single country on a single ethnic group and the different quartile used in each study, it is not possible at this stage to provide a specific cut-off for increased risk of GDM based on RBC folate levels. Furthermore, there were several other variations in the included studies like, the difference in adjusted confounding factors, time of measurement of folate levels, the ethnicity of the population, use of folate supplements, and classification of data, all of which could have contributed to the varied results amongst studies especially for the association of serum folate levels and risk of GDM. In the two studies reporting an increased risk of GDM with serum folate levels, the effect size was small with the lower end of 95% CI close to 1 [(25): 1; (23): 1.01]. Also, in two of the four studies reporting no relationship between serum folate and GDM, the sample size was small and this could have affected the results (20, 26).

Our analysis also demonstrated lower vitamin B12 levels in the GDM group as compared to the non-GDM group. Vitamin B12 deficiency is conventionally diagnosed if serum B12 levels are <203.3 pg/ml (150 pmol/L) (34). In our review, Sukumar et al. (22) and Krishnaveni et al. (20) both demonstrated an increased risk of GDM with vitamin B12 deficiency. In concurrence with these studies, Li et al. (25) found that vitamin B12 levels higher than 234 pg/ml are associated with a significantly reduced risk of GDM. Similar results were noted by Lai et al. (23). However, contrasting results were demonstrated by Chen et al. (13), who reported higher vitamin B12 levels to be associated with an increased risk of GDM. The variation of results may be explained by the timing of serum vitamin B12 measurements in the included studies. While all four studies reporting an inverse relationship between vitamin B12 levels and risk of GDM measured B12 levels in the second or third trimester of pregnancy, Chen et al. (13) measured the same at early pregnancy (9–13 weeks). Studies indicate physiological changes of pregnancy may affect vitamin B12 requirement with a decrease in vitamin B12 levels from preconception to mid-gestation (35, 36). Since both vitamin B12 and folic acid are closely related to nucleic acid synthesis, methyl group generation, and conversion of homocysteine to methionine; the possibility of one micronutrient confounding the effects of another cannot be ruled out (13). Important to note is that three of the four studies demonstrating a higher risk of GDM with low vitamin B12 reported adjusted ORs with folate as a dependent variable.

The mechanism of increased risk of GDM with folate and vitamin B12 imbalance is, however, unclear. One explanation is high folate aggravates the effects of vitamin B12 deficiency, which in turn impairs DNA synthesis particularly mitochondrial DNA, leading to the development of insulin resistance (20, 37). Secondly, high levels of unmetabolized serum folate are known to reduce the cytotoxicity of natural killer cells (38). The reduction of cytotoxicity has also been implicated in the pathogenesis of GDM (39).

Our study has some limitations. Firstly, a limited number of studies were available for a meta-analysis. Pooled analysis for RBC folate, serum folate, and serum vitamin B12 as continuous variables could include just three studies. Due to the difference in quartiles in the included studies, only a descriptive analysis could be carried out. Four studies did not report adjusted ORs for risk of GDM. Furthermore, there was wide variation amongst the studies in the factors adjusted in the multi-variable analysis. Overall, age, parity, vitamin B12/folate levels and body mass index were the most common factors adjusted but were not coherent across studies. Such differences could have influenced the outcomes of individual studies. Secondly, there were wide variations in the included studies about the time of testing and criteria for the diagnosis of GDM which could have skewed results. Thirdly, the majority of the included studies included only a small number of GDM patients (<50). Lack of adequate power may have influenced the results. Lastly, the majority of studies were from Asian countries and generalization of the findings to other ethnic populations requires further research.

To conclude, the association between vitamin B12 and folate levels during pregnancy and the risk of GDM is not very clear. Unadjusted data is indicative of lower vitamin B12 levels in women with GDM as compared to their healthy counterparts, but folate levels are not different. Limited number of studies indicate increased risk of GDM with higher RBC folate levels, but majority studies found no association between serum folate and risk of GDM. Based on available studies, the association between the risk of GDM with vitamin B12 deficiency is conflicting. There is a need for further large-scale studies from different regions worldwide to strengthen current evidence.

## Data Availability Statement

Publicly available datasets were analyzed in this study. This data can be found here: PubMed, Embase, CENTRAL, and Ovid databases.

## Author Contributions

LW conceived, designed the study, and wrote the paper. YH, DM, LY, and XM were involved in literature search, data collection, and analyzed the data. FL reviewed and edited the manuscript. All authors read and approved the final manuscript.

## Conflict of Interest

The authors declare that the research was conducted in the absence of any commercial or financial relationships that could be construed as a potential conflict of interest.

## Supplementary Material

Supplementary Figure 1 Funnel plot for the meta-analysis serum folate concentration during pregnancy between GDM and non-GDM groups.

Supplementary Figure 2 Funnel plot for the meta-analysis of serum vitamin B12 concentration during pregnancy between GDM and non-GDM groups.

Supplementary Figure 3 Funnel plot for the meta-analysis of adjusted odds ratios assessing relationship between GDM and RBC folate (as continuous variable).

Supplementary Figure 4 Funnel plot for the meta-analysis of adjusted odds ratios assessing relationship between GDM and serum folate (as continuous variable).

Supplementary Figure 5 Meta-analysis of adjusted odds ratios assessing relationship between GDM and vitamin B12 (as continuous variable).

Supplementary Table 1 Search strategy and results of PubMed database.

Supplementary Table 2 Risk of bias analysis.
