# Supplementary figures and images for: Vitamin B12 and Folate Levels During Pregnancy and Risk of Gestational Diabetes Mellitus: A Systematic Review and Meta-Analysis
Source: Front Nutr. 2021 Jun 14;8:670289. doi: 10.3389/fnut.2021.670289 (PMC8236507; doi:10.3389/fnut.2021.670289)

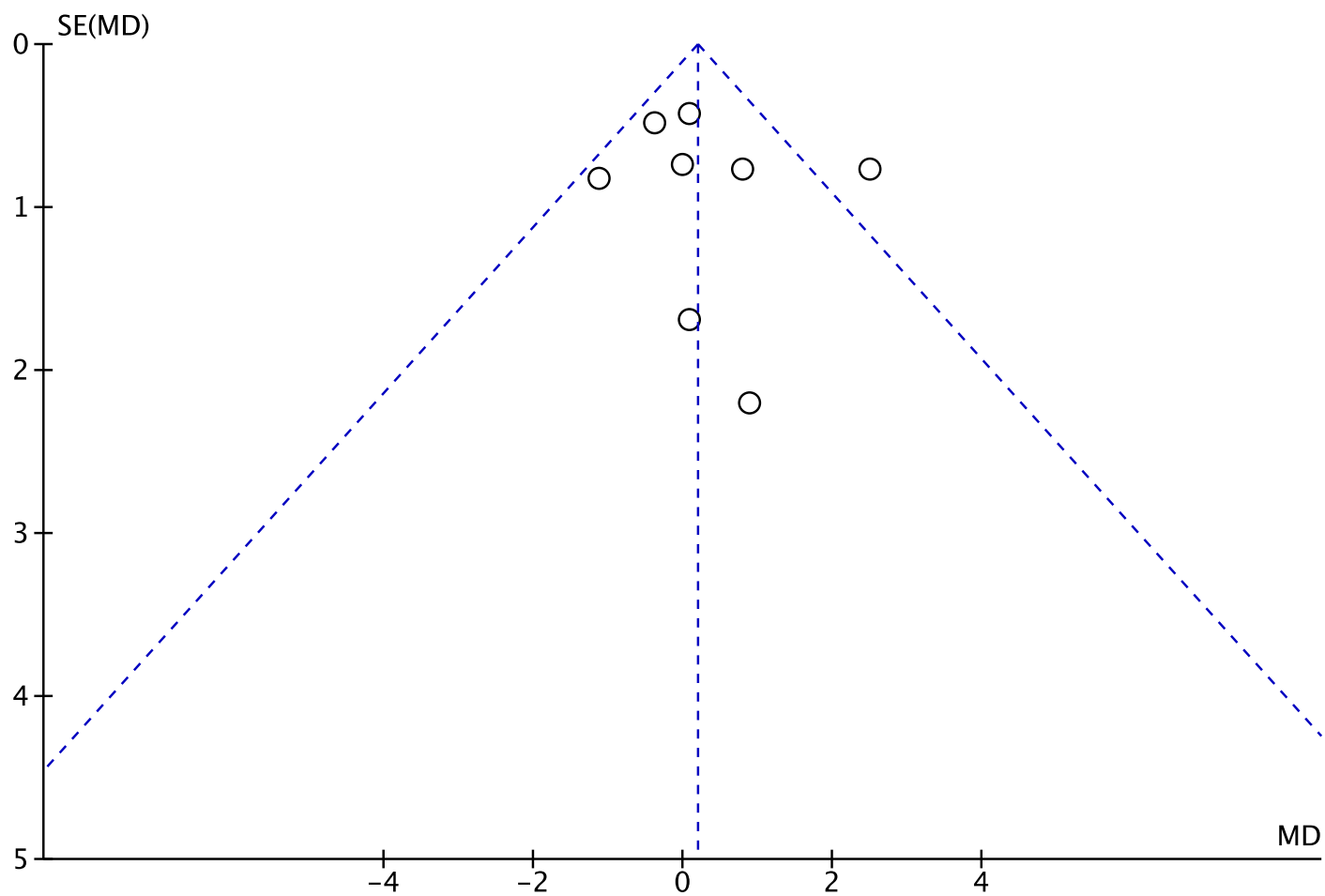

Supplement: Supplementary Figure 1 — Funnel plot for the meta-analysis serum folate concentration during pregnancy between GDM and non-GDM groups. [file Data_Sheet_1.PDF]

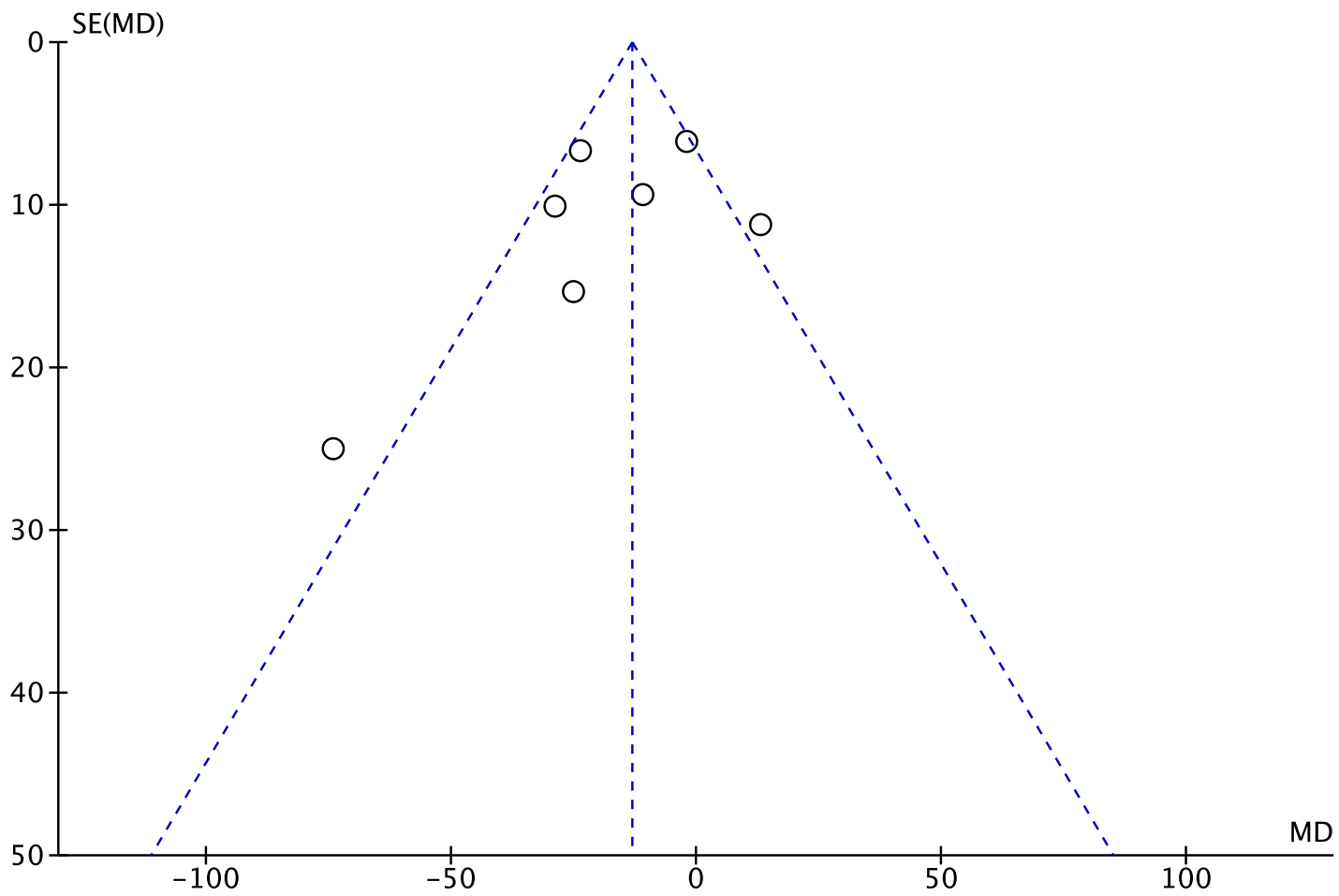

Supplement: Supplementary Figure 2 — Funnel plot for the meta-analysis of serum vitamin B12 concentration during pregnancy between GDM and non-GDM groups. [file Data_Sheet_2.PDF]

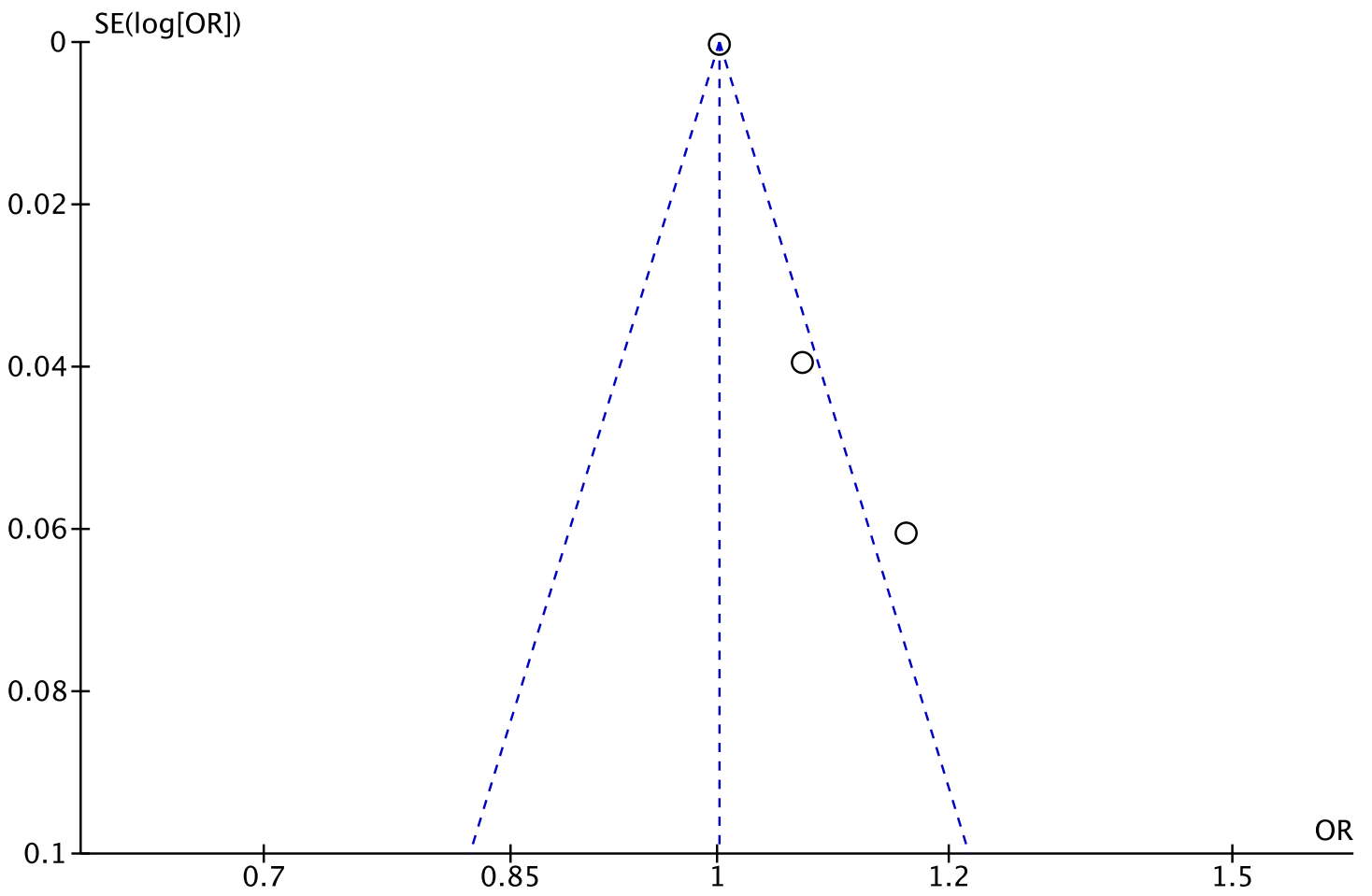

Supplement: Supplementary Figure 3 — Funnel plot for the meta-analysis of adjusted odds ratios assessing relationship between GDM and RBC folate (as continuous variable). [file Data_Sheet_3.PDF]

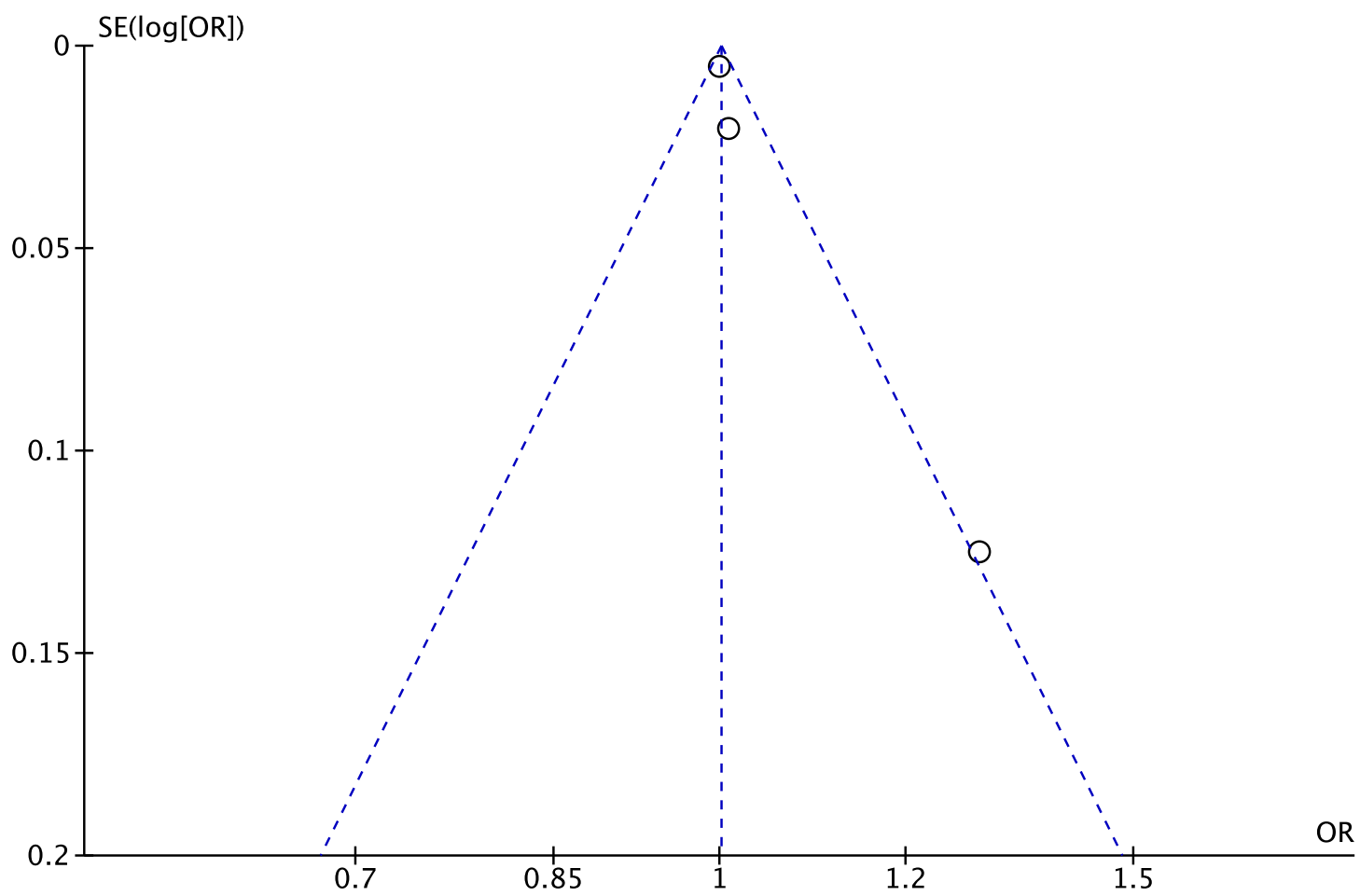

Supplement: Supplementary Figure 4 — Funnel plot for the meta-analysis of adjusted odds ratios assessing relationship between GDM and serum folate (as continuous variable). [file Data_Sheet_4.PDF]

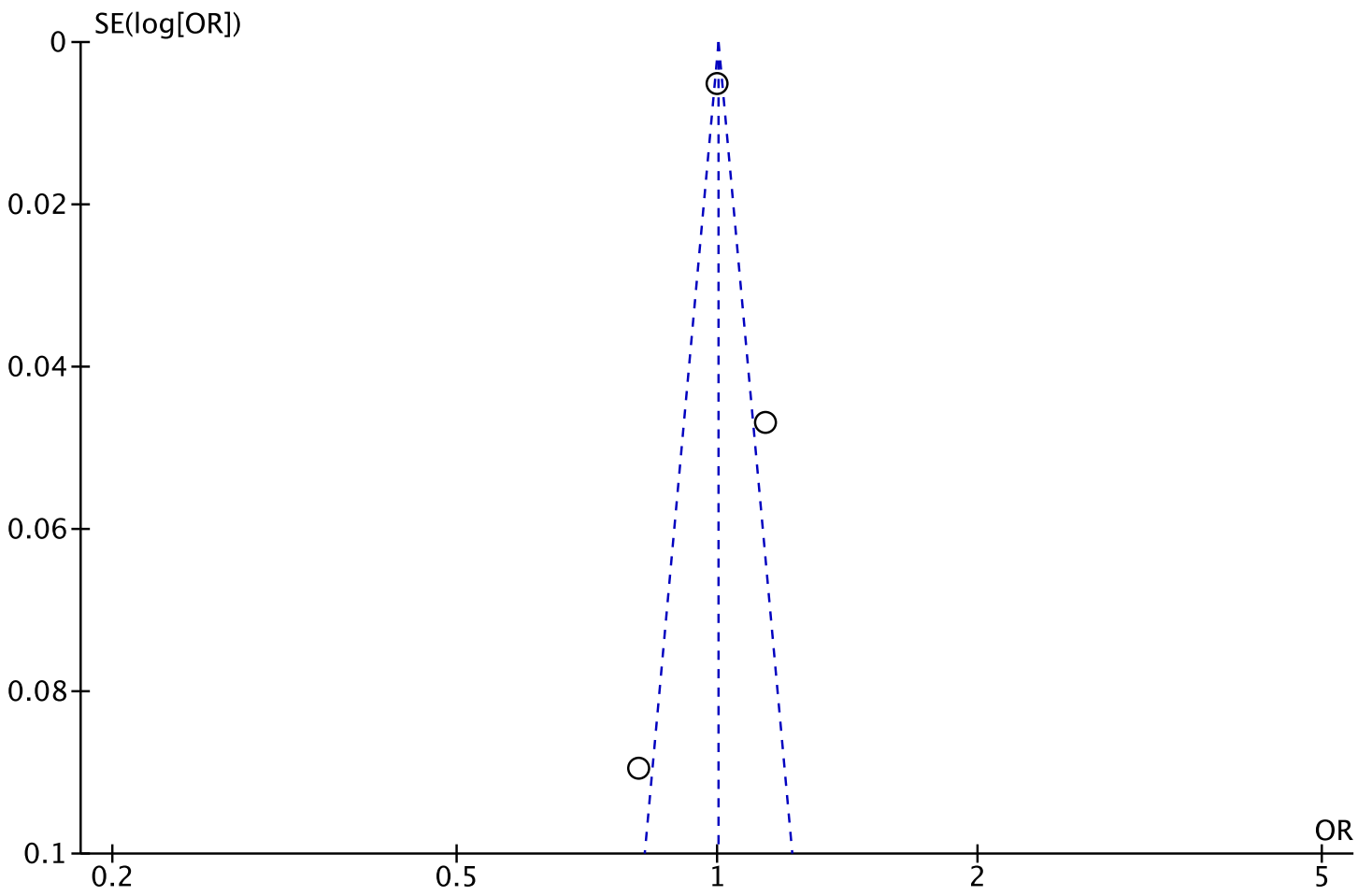

Supplement: Supplementary Figure 5 — Meta-analysis of adjusted odds ratios assessing relationship between GDM and vitamin B12 (as continuous variable). [file Data_Sheet_5.PDF]
