# Supplementary material for: Vitamin B12 and Folate Levels During Pregnancy and Risk of Gestational Diabetes Mellitus: A Systematic Review and Meta-Analysis
Source: Front Nutr. 2021 Jun 14;8:670289. doi: 10.3389/fnut.2021.670289 (PMC8236507; doi:10.3389/fnut.2021.670289)
Supplement: Supplementary Table 1 — Search strategy and results of PubMed database. [file Table_1.DOCX]

Supplementary Table 1. Search strategy and results of PubMed database

| **Query** | **Search Details** | **Results** |
| --- | --- | --- |
| ((folate) AND (gestational)) AND (hyperglycaemia) | ("folic acid"[MeSH Terms] OR ("folic"[All Fields] AND "acid"[All Fields]) OR "folic acid"[All Fields] OR "folate"[All Fields] OR "folates"[All Fields]) AND ("gestate"[All Fields] OR "gestated"[All Fields] OR "gestates"[All Fields] OR "gestating"[All Fields] OR "gestational"[All Fields] OR "gestations"[All Fields] OR "pregnancy"[MeSH Terms] OR "pregnancy"[All Fields] OR "gestation"[All Fields]) AND ("hyperglycaemia"[All Fields] OR "hyperglycaemia"[MeSH Terms] OR "hyperglycaemia"[All Fields] OR "hyperglycaemias"[All Fields] OR "hyperglycaemias"[All Fields] OR "hyperglycaemia s"[All Fields]) | 22 |
| ((folic acid) AND (gestational)) AND (hyperglycaemia) | ("folic acid"[MeSH Terms] OR ("folic"[All Fields] AND "acid"[All Fields]) OR "folic acid"[All Fields]) AND ("gestate"[All Fields] OR "gestated"[All Fields] OR "gestates"[All Fields] OR "gestating"[All Fields] OR "gestational"[All Fields] OR "gestations"[All Fields] OR "pregnancy"[MeSH Terms] OR "pregnancy"[All Fields] OR "gestation"[All Fields]) AND ("hyperglycaemia"[All Fields] OR "hyperglycaemia"[MeSH Terms] OR "hyperglycaemia"[All Fields] OR "hyperglycaemias"[All Fields] OR "hyperglycaemias"[All Fields] OR "hyperglycaemia s"[All Fields]) | 17 |
| ((Vitamin B12) AND (gestational)) AND (hyperglycaemia) | ("vitamin b 12"[MeSH Terms] OR "vitamin b 12"[All Fields] OR ("vitamin"[All Fields] AND "b12"[All Fields]) OR "vitamin b12"[All Fields]) AND ("gestate"[All Fields] OR "gestated"[All Fields] OR "gestates"[All Fields] OR "gestating"[All Fields] OR "gestational"[All Fields] OR "gestations"[All Fields] OR "pregnancy"[MeSH Terms] OR "pregnancy"[All Fields] OR "gestation"[All Fields]) AND ("hyperglycaemia"[All Fields] OR "hyperglycaemia"[MeSH Terms] OR "hyperglycaemia"[All Fields] OR "hyperglycaemias"[All Fields] OR "hyperglycaemias"[All Fields] OR "hyperglycaemia s"[All Fields]) | 6 |
| (folic acid) AND (gestational diabetes) | ("folic acid"[MeSH Terms] OR ("folic"[All Fields] AND "acid"[All Fields]) OR "folic acid"[All Fields]) AND ("diabetes, gestational"[MeSH Terms] OR ("diabetes"[All Fields] AND "gestational"[All Fields]) OR "gestational diabetes"[All Fields] OR ("gestational"[All Fields] AND "diabetes"[All Fields])) | 132 |
| (folate) AND (gestational diabetes) | ("folic acid"[MeSH Terms] OR ("folic"[All Fields] AND "acid"[All Fields]) OR "folic acid"[All Fields] OR "folate"[All Fields] OR "folates"[All Fields]) AND ("diabetes, gestational"[MeSH Terms] OR ("diabetes"[All Fields] AND "gestational"[All Fields]) OR "gestational diabetes"[All Fields] OR ("gestational"[All Fields] AND "diabetes"[All Fields])) | 168 |
| (Vitamin B12) AND (gestational diabetes) | ("vitamin b 12"[MeSH Terms] OR "vitamin b 12"[All Fields] OR ("vitamin"[All Fields] AND "b12"[All Fields]) OR "vitamin b12"[All Fields]) AND ("diabetes, gestational"[MeSH Terms] OR ("diabetes"[All Fields] AND "gestational"[All Fields]) OR "gestational diabetes"[All Fields] OR ("gestational"[All Fields] AND "diabetes"[All Fields])) | 45 |
