# Supplementary material for: Vitamin B12 and Folate Levels During Pregnancy and Risk of Gestational Diabetes Mellitus: A Systematic Review and Meta-Analysis
Source: Front Nutr. 2021 Jun 14;8:670289. doi: 10.3389/fnut.2021.670289 (PMC8236507; doi:10.3389/fnut.2021.670289)
Supplement: Supplementary Table 2 — Risk of bias analysis. [file Table_2.DOCX]

Supplementary Table S2. Risk of bias analysis

| Study | Selection of participants | Confounding variables | Measurement of exposure | Blinding of outcome assessment | Incomplete outcome data | Selective outcome reporting |
| --- | --- | --- | --- | --- | --- | --- |
| Jankovic-Karasoulos 2020 [18] | Low risk | Low risk | Low risk | High risk | Unclear risk | Low risk |
| Liu 2020 [19] | Low risk | Low risk | Low risk | High risk | Unclear risk | Low risk |
| Chen 2020 [13] | Low risk | Low risk | Low risk | High risk | Unclear risk | Low risk |
| Xie 2019 [26] | Low risk | Low risk | Low risk | High risk | Unclear risk | Low risk |
| Li 2019 [27] | Low risk | Low risk | Low risk | High risk | Unclear risk | Low risk |
| Lai 2017 [25] | Low risk | Low risk | Low risk | High risk | Unclear risk | Low risk |
| Sukumar 2016[24] | Low risk | Low risk | Low risk | High risk | Unclear risk | Low risk |
| Krishnaveni 2009[22] | Low risk | Low risk | Low risk | High risk | Unclear risk | Low risk |
| Idzior-Walus 2008[23] | Low risk | High risk | Low risk | High risk | Unclear risk | Low risk |
| Guven 2006[21] | Low risk | High risk | Low risk | High risk | Unclear risk | Low risk |
| Tarim 2004[20] | Low risk | High risk | Low risk | High risk | Unclear risk | Low risk |
| Seghieri 2003[17] | Low risk | High risk | Low risk | High risk | Unclear risk | Low risk |
